# Food and Grain Consumption Per Capita in the Qinghai–Tibet Plateau and Implications for Conservation

**DOI:** 10.3390/nu13113742

**Published:** 2021-10-23

**Authors:** Lijing Wang, Yi Xiao, Zhiyun Ouyang

**Affiliations:** 1 State Key Laboratory of Urban and Regional Ecology, Research Center for Eco-Environmental Sciences, Chinese Academy of Sciences, Beijing 100085, China; wanglijingcas@163.com (L.W.); xiaoyi@rcees.ac.cn (Y.X.); 2 University of Chinese Academy of Sciences, Beijing 100049, China

**Keywords:** nutrient, per capita grain consumption, grain security, Qinghai–Tibet Plateau

## Abstract

Background: Grain security is crucial for social stability and ecosystem conservation regionally and globally, and it is particularly concerned widely in the Qinghai-Tibet Plateau (QTP) due to its high altitude and harsh climate for agriculture. Method: In this paper, we calculated and analyzed per capita food and grain consumption, including direct grain consumption, grain for fodder, industry consumption, seeds consumption, and wastage consumption and its changes in the QTP during 1995–2019. Results: The results showed that (1) in 2019, the average food consumption per capita was 333.35 kg, was stable since 1995. The dietary structure of residents was composed of direct grain consumption (44.15%), meat (10.72%), and milk (6.94%). The consumption of meat and milk was higher than the national average. (2) The average daily intake of energy and protein, animal protein, and the ratio of high-quality protein and fat energy were 2156.21 kcal·d^−1^, 73.53 g·d^−1^, 23.06 g·d^−1^, 38.32%, and 27.77% in 2019. Their changes were −342.98 kcal·d^−1^, −8.91 g·d^−1^, 11.16 g·d^−1^, 18.37%, and 11.08%, respectively. (3) The corresponding grain consumption per capita was 284.90 kg·a^−1^ in 1995, 262.19 kg·a^−1^ in 2010, and then remained stable until 2019. Conclusion: The study suggested that food consumption per capital was guaranteed at the well-off level since 2010, and food and dietary structure of residents were corresponding to physical geographic and climatic environment in the QTP. The conflict between food security and the ecosystem conservation can be managed without scarifying nature as the total grain consumption was stable since 2010, and the yield per unit area and total grain yield were both increasing since 2003 for agricultural condition improved in the QTP.

## 1. Introduction

Grain security is very important to residents’ wellbeing, social stability, and regional economic development [1,2,3]. Grain consumption per capita is the basic index to evaluate grain security from the demand side, which is of great significance for research on regional grain security for their abundant nutrients [4,5]. At present, grain consumption per capita has been studied from five aspects: direct grain consumption, grain for fodder, industry consumption, seeds consumption, and wastage consumption [6,7]. The dietary habits of residents in various regions are different, although the demand in nutrients for humans is almost the same, so we can calculate the corresponding grain consumption. Tang suggested that the maximum per capita grain demand in China under the dietary balance mode was 386.6 kg·a^−1^ [8]. While, Xie believed it was 384 kg·a^−1^ based on nutrients’ demand in the new era [9]. The per capita grain demand standard in China was 400 kg as the nutrient intake standard of the well-off level [10]. The standard has been an important reference in policy making for national and regional grain production and security in China [1,11,12,13,14,15,16].

Due to its unique geographical conditions, ecosystems, population composition, and dietary habits, the grain security in the QTP has attracted extensive attention [17,18,19]. The dietary structure of residents in the QTP is different from the nationwide average of China. The QTP is the highest-altitude region in the world. There are few land resources suitable for cultivated land, but it is more suitable for forage. The dominant ecosystem type in the QTP is the grassland ecosystem [20], which is essential for development of animal husbandry. Correspondingly, the residents in the QTP had higher consumption of beef, mutton, and milk, and lower consumption of vegetables and aquatic products than the residents in other regions. The main energy, protein, and fat obtained by Tibetan in Qinghai Province come from animals. Mongolia and Kazak also have a similar situation [10].

There was a long debate regarding whether the population in Tibet was overloaded or not, it was a challenge for ecosystem protection and restoration programs. Hao and others discovered that the population of the Tibet was not overloaded [21]. The conclusion was opposite to the previous research results [22,23]. Therefore, it is necessary to analyze grain consumption that is suitable for the QTP to evaluate the grain security in this region.

We studied the per capita food and grain consumption through direct grain consumption, grain for fodder, industry consumption, seeds consumption, and wastage consumption from the perspective of nutritional demand in the QTP from 1995 to 2019. The purpose of this study was to explore how food and dietary structure of residents correspond to physical geographic and climatic environment, and test whether the conflict between food security and the ecosystem conservation can be managed.

## 2. Material and Methods

### 2.1. Study Area

The QTP is a unique geological–geographical–ecological unit [24], located in the interior of Asia, and known as the “Roof of the World”, with an average altitude of more than 4000 m. Its total area is about 2.56 million km^2^ [25]. Qinghai and Tibet are located in the core of the QTP, with a total population of 9.58 million in 2018. There were 5.76 million Tibetans, accounting for 60.10% of the total population. Taking the two provinces as the representatives of the QTP, this study analyzed the per capita food and grain consumption in this region.

### 2.2. Method

#### 2.2.1. Per Capita Grain Consumption

The food in this study include direct grain consumption, edible oil (vegetable oil and animal oil), meat (pork, beef, and mutton), poultry, aquatic products, eggs, dairy, vegetables, fruits, sugar and alcohol, the grains include cereals, beans, and tubers. Additionally, the grain-consuming animal products include animal oil, meat, poultry, aquatic products, eggs, and dairy, so they can be transformed into grain for fodder, the same with alcohol.

In this study, according to the production sources of food consumed by residents, the grain consumption was divided into five types: direct grain consumption, grain for fodder, industry consumption, seed consumption, and wastage consumption (Figure S1). The grain for fodder, industry consumption, seed consumption, and wastage consumption are all indirect grain consumption. The sum of the five types of grain was the per capita grain consumption. The per capita grain consumption was calculated using the following formula [8]:

$$G=\sum_{i=1}^{n}g_{i}$$

where G was the per capita grain consumption and $g_{i}$ was the i type grain consumption. In this paper, the weighted average of Qinghai and Tibet provinces represents the per capita grain consumption in the QTP.

#### 2.2.2. Direct Grain Consumption

The direct grain consumption was calculated using the following formula [8]:

$$D=\sum_{i=1}^{n}P_{i}\cdot \left(BD_{i}+OD_{i}\right)$$

where D was per capita direct grain consumption, $P_{i}$ was the population proportion of the i region, $BD_{i}$ was the per capita direct grain consumption purchase or consumption at home in the i region, and $OD_{i}$ was the per capita direct grain consumption out of home in the i region. In this study, there were two regions, namely, urban and rural.

#### 2.2.3. Grain for Fodder

The grain for fodder was calculated using the following formula [8]:

$$F=\sum_{i=1}^{n}\sum_{j=1}^{n}\left(\frac{BA_{ij}+OA_{ij}}{1-L_{j}}\cdot C_{j}\right)$$

where F was per capita grain for fodder, $BA_{ij}$ was the consumption of the j animal product in the i region at home, $OA_{ij}$ was the consumption of the j animal product in the i region out of home, $L_{j}$ was the loss rate of the i animal product, and $C_{j}$ was the grain consumption coefficient of the j animal product. There were seven animal products in this study, namely, pork, beef, mutton, poultry, eggs, milk, and aquatic products.

#### 2.2.4. Industry Consumption

The industry consumption was calculated using the following formula [8]:

$$IN=\sum_{i=1}^{n}\sum_{j=1}^{n}\left[\left(BW_{ij}+OW_{ij}\right)\cdot C_{j}\right]$$

where IN was industry consumption, $BW_{ij}$ was the per capita consumption of the j industrial product in the i region at home,$\;OW_{ij}$ was the per capita consumption of the j industrial product in the i region out of home. $C_{j}$ was the grain consumption coefficient of the j industrial product. There were two industrial products in this study, namely, Chinese Baijiu and beer.

#### 2.2.5. Seeds Consumption

The seeds consumption was calculated using the following formula [8]:

$$S=G_{p}\cdot SR$$

where S was seed consumption, $G_{p}$ was the per capita grain yield, and SR was the proportion of seed grain.

#### 2.2.6. Wastage Consumption

The wastage consumption was calculated using the following formula [8]:

$$WA=G_{p}\cdot WR$$

where WA was the wastage consumption and WR was the grain loss rate.

### 2.3. Data Sources

#### 2.3.1. Food Consumption at Home

The annual average number of commodities purchased by urban households, the average consumption of main food by rural households, and the urban and rural population were derived from the Qinghai Statistical Yearbook [26] and the Tibet Statistical Yearbook [27].

#### 2.3.2. Food Consumption out of Home

The proportion of residents dining out of home comes from the survey data of the China chronic disease surveillance questionnaire [28], the food composition of residents dining out comes from the data of the China Health and Nutrition Survey [29], the expenditure of urban residents dining out of home comes from the Qinghai Statistical Yearbook [26] and the Tibet Statistical Yearbook [27], and the expenditure of rural residents on dining out of home comes from the Yearbook of China’s rural household survey [30]. The data for other years were interpolated by the consumption ratio of urban and rural residents [31].

#### 2.3.3. Direct Grain Consumption

Direct grain consumption was calculated when we collected the data in Section 2.3.1 and Section 2.3.2.

#### 2.3.4. Grain for Fodder

Based on the research on grain for fodder by Han [32] and others, combined with the loss rate and grain consumption coefficient of animal products [9,33,34] at a similar time, the loss rate and grain consumption coefficient of animal products were formulated.

#### 2.3.5. Industry Consumption

Industry consumption included the grain used in Chinese Baijiu and beer. The data were from the Qinghai Statistical Yearbook [26] and the Tibet Statistical Yearbook [27]. The grain consumption coefficient was obtained by referring to the research results of Tang [8] and Shen [35].

#### 2.3.6. Seeds Consumption

The sowing area of grain and the average sowing data per unit area were from the China Statistical Yearbook [36] and the national compilation of cost–benefit data of agricultural products [37]. The national proportion of seed consumption was taken as the standard of the QTP.

#### 2.3.7. Wastage Consumption

The grain loss rate was estimated as 4% in this paper, according to the actual situation in the process of grain transportation, storage, and management [38].

#### 2.3.8. Nutrient Content of Foods

The nutrient content of foods comes from the China Food Composition Tables Standard Edition [39,40]. The proportion of food composition referred to the research of China’s Medium and Long Term Food Development Strategy [10] and Gao [19] and Liu [17] (Table S1).

#### 2.3.9. Well-Off Nutritional Level

According to the well-off nutritional level proposed in China’s Medium and Long Term Food Development Strategy [10] and Chinese Dietary Reference Intakes proposed in the dietary nutrient reference intake of Chinese residents [41], the well-off nutritional level in this study was proposed: the daily per capita energy intake is 2100 kcal·d^−1^ and the protein is 70 g·d^−1^, of which the high-quality protein accounts for more than 30% and animal protein accounts for more than 20%, the ratio of energy from fat is 20–25%.

## 3. Results

We calculated and analyzed per capita food and grain consumption, and nutrient intakes in the QTP during 1995–2019. The grain consumption included direct grain consumption, grain for fodder, industry consumption, seeds consumption, and wastage consumption.

### 3.1. Food Consumption Per Capita and Nutrient Intakes

**Figure 1:**
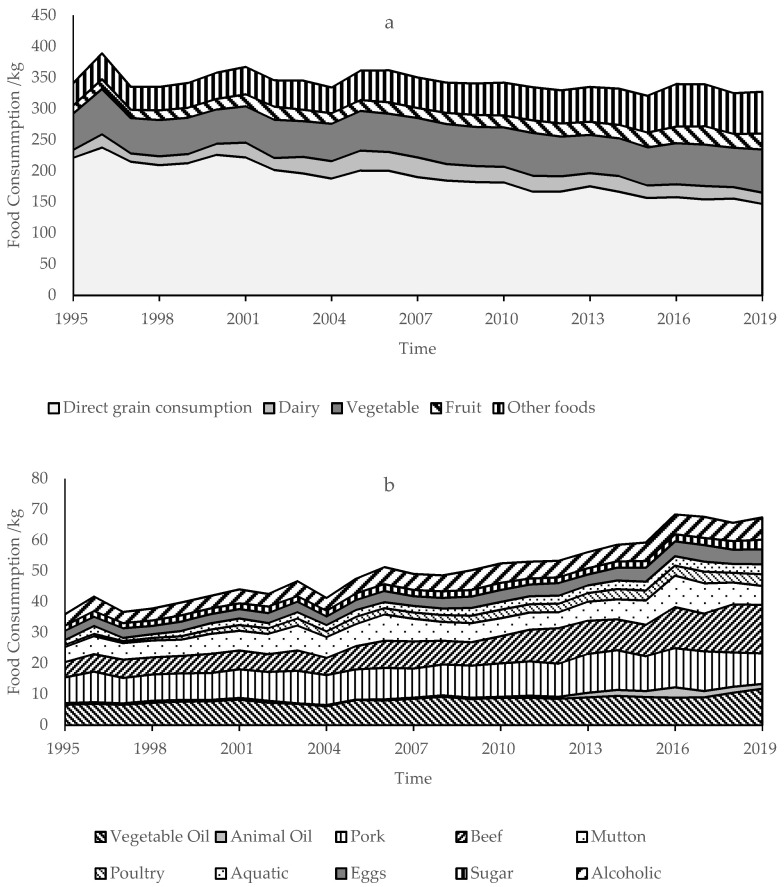
The structure of per capita food consumption in the QTP from 1995 to 2019 (**a**,**b**). The other food in a was displayed in b to show them clearly. The foods in (**b**) were far less than direct grain consumption, dairy, vegetable and fruit, so they were not clear in (**a**).

The total per capita food consumption in the QTP was generally stable, but the food structure was greatly changed from 1995 to 2019 (Figure 1). The total food consumption in the QTP was 333.35 kg in 2019. Its multi-year average was 333.77 kg, and the anomalous percentage of each year was less than 8% (except for 1996). Among all types of food, the consumption of direct grain consumption declined from 221.01 to 147.17 kg. The consumption of meat, such as pork, poultry, beef and mutton, was 9.99, 3.83, and 21.90 kg in 2019, respectively. The consumption of pork only increased by 18.53%, but the consumption of beef and mutton increased by 123.42%. The proportion of beef and mutton had increased, and the proportion of pork had decreased year by year. Milk consumption was 23.14 kg in 2019 and increased by 77.05%. Edible oil, poultry, aquatic products, eggs, vegetables, fruits, sugars, and alcohol also increased to some extent.

**Figure 2:**
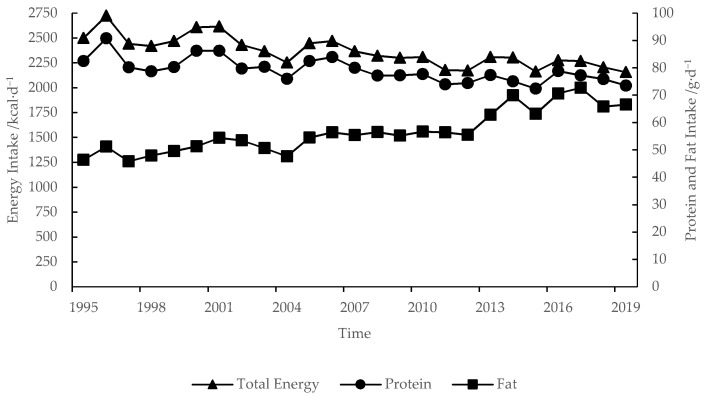
Per capital-nutrient intake in the QTP from 1995 to 2019.

**Figure 3:**
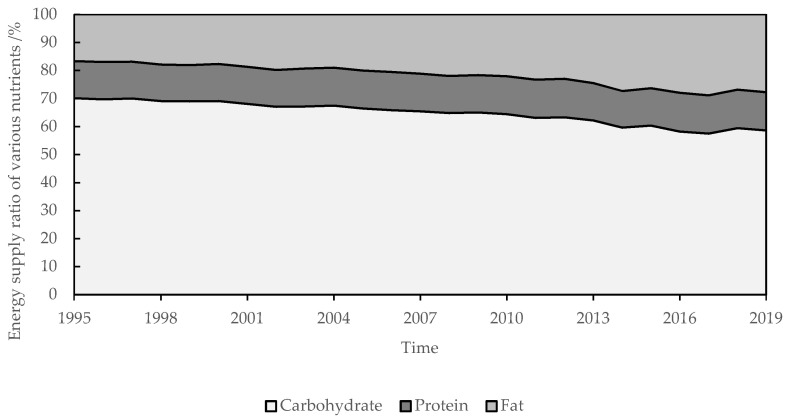
Per capita energy supply ratio of various nutrients in the QTP from 1995 to 2019.

**Figure 4:**
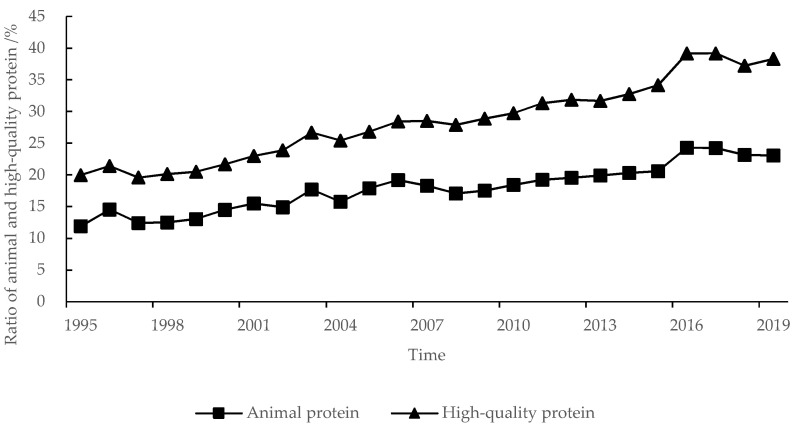
Ratio of animal and the high-quality protein in the QTP from 1995 to 2019.

The per capita nutrient intake and composition in the QTP was intensively changed from 1995 to 2019. The energy intake decreased from 2499.19 to 2156.21 kcal·d^−1^ (Figure 2), in which the ratio of energy from carbohydrates decreased from 70.11% to 58.59%, in contrast, the ratio of energy from fat increased from 16.69% to 27.77%, and the ratio of energy from protein basically remained at about 13.40% (Figure 3). Protein intake was 73.53 g·d^−1^, and decreased by 8.91 g·d^−1^, but the ratio of animal protein and the high-quality protein increased from 17.44% and 19.95% to 31.36% and 38.32%, respectively (Figure 4). Fat intake was 66.53 g·d^−1^ and increased by 20.18 g·d^−1^.

In 2010, per capita nutrient intake of residents in the QTP reached the well-off nutritional level. Before 1995, the average daily intake of energy and protein had exceeded 2100 kcal·d^−1^ and 70 g·d^−1^. In 2003, the average daily intake of animal protein had exceeded 20%. In 2010, the ratio of high-quality protein and energy from fat had exceeded 30% and 20%.

### 3.2. Per Capita Grain Consumption and Its Change

**Figure 5:**
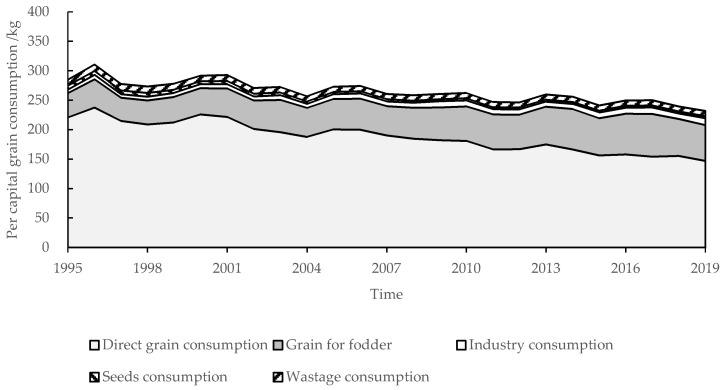
The structure of per capita grain consumption in the QTP from 1995 to 2019.

The per capita grain consumption in the QTP decreased by 24.16 kg before 2007, and then remained stable (Figure 5), the anomalous percentage of each year was less than 5% (except in 2019). Among them, direct grain consumption decreased by 73.84 kg from 1995 to 2019 (Figure 1 and Figure 5), which was the main reason for the reduction of grain consumption. However, it was still the main part of grain consumption, accounting for 63.47% in 2019. The indirect grain consumption was 84.69 kg in 2019, increased by 20.67 kg from 1995 to 2019. Among all types of indirect grain consumption, grain for fodder, industry consumption, seed consumption, and wastage consumption were 60.90, 11.75, 3.26, and 8.78 kg in 2019, respectively. The grain for fodder increased by 19.91 kg. The proportion of grain for fodder increased from 14.39% to 26.26%. The industry consumption increased by 4.29 kg, and the seed consumption and wastage consumption decreased by 3.25 and 1.38 kg, respectively. The proportion of their sum increased from 8.08% to 10.26%. The industry consumption, seed consumption and wastage consumption were always less than the direct grain consumption and grain for fodder.

In this study, the maximum per capita grain consumption after 2010 was taken as the per capita grain consumption of the well-off nutritional level in the QTP, i.e., 262 kg·a^−1^.

### 3.3. Comparison of Per Capita Grain Consumption between the QTP and Nationwide

**Figure 6:**
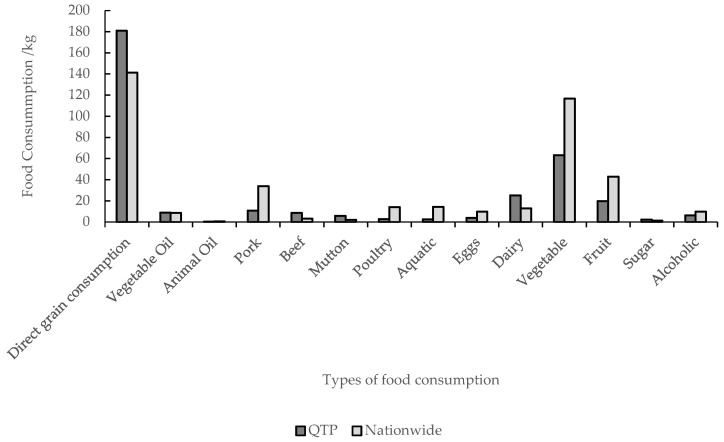
Per capita food consumption in the QTP and nationwide.

**Figure 7:**
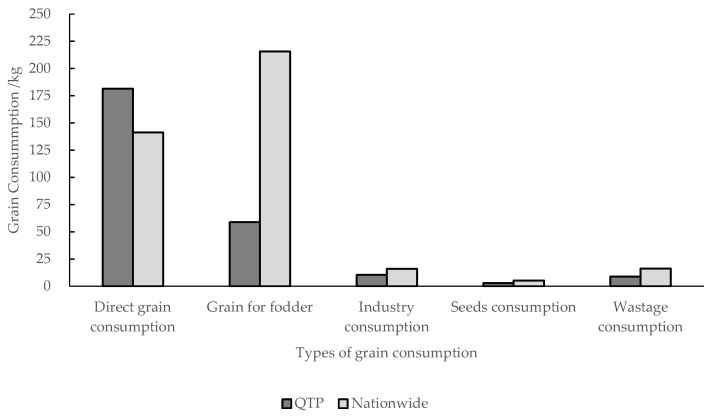
Per capita grain consumption of the well-off nutritional level in the QTP and nationwide.

The difference in dietary structure was the main reason for the large difference in grain consumption between the QTP and the nationwide well-off nutritional level (Figure 6). The main differences were direct grain consumption and animal food (grain for fodder). The differences between direct grain consumption and grain for fodder were 39.65 and 157.25 kg (Figure 7). This is because the residents in the QTP consumed more non-grain-consuming animal products, such as beef, mutton, and milk, and less grain-consuming animal products, such as pork, poultry, eggs, and aquatic products than the residents in other regions. The difference of other types of grain consumption was relatively small.

## 4. Discussion and Conclusions

Based on the per capita food consumption of residents in the QTP from 1995 to 2019, we studied the per capita grain consumption and its change trend from the perspective of nutritional demand. Our research showed the following:

(1) Food and dietary structure of residents corresponded to physical geographic and climatic environment. The QTP is one of the traditional pastoral areas in China, food and dietary structure of residents have obvious regional characteristics, and they are still retained in modern society. The consumption of beef and mutton was high and exceeded the recommended value in the reasonable dietary pagoda of Chinese residents. The consumption of edible oil was too high, so we need to be vigilant against the occurrence of various cardiovascular and cerebrovascular diseases. The consumption of milk was also higher than that in non-pastoral regions, but it had not met the recommended standards, and a similar situation had occurred in the Hulunbuir pastoral area [42]. Among all types of food, only cereals and tubers met the recommended value standard. Cereals and tubers were the main foods, accounting for 44.26% of the total food consumption, which was different from the previous impression of the food consumption structure of “non-staple food (meat, milk and vegetables) dominated” of pastoral residents [42,43]. Other types of food did not meet the recommended standards (Table 1). Meanwhile, for vegetables and fruits, the QTP had never been able to feed itself, over 75% vegetables and fruits had to be transported from other provinces, so the grain security can be regarded as food security in the QTP.

(2) In 2010, residents in the QTP met all indicators of the well-off nutritional level. Due to the change of dietary structure of residents, energy intake decreased from 2451.61 kcal·d^−1^ in 1995 to 2309.52 kcal·d^−1^ in 2010, and then to 2156.21 kcal·d^−1^ in 2019, which was consistent with the changing trend of national per capita energy intake [44,45,46,47]. The reference value of per capita energy intake in the Chinese Dietary Reference Intakes also decreased from 2254.89 kcal·d^−1^ in 2000 to 2038.58 kcal·d^−1^ in 2013 [41]. Beef and mutton have lower energy, lower fat, and higher protein than pork [40]. In addition, residents consumed a lot of beef, mutton, and milk. Therefore, the protein intake of residents had exceeded 70 g·d^−1^ before 1995. With the reduction of direct grain consumption and the increase of animal products consumption, the proportion of plant protein decreased, meanwhile, the proportion of animal protein and the high-quality protein was increased year by year.

(3) The conflict between food security and the ecosystem conservation can be managed without scarifying nature as the total grain consumption was stable since 2010 and yield per unit area and total grain yield were both increasing in the QTP. The per capita grain consumption of the well-off nutritional level in the QTP was 262 kg·a^−1^, and stable since 2010, which was related to the mode of animal husbandry in this area. The QTP is an important practice area of “store grain in grassland”. With the implementation of ecosystem protection and restoration programs, such as “Grain for Green”, the western region fully developed animal husbandry, reformed agricultural structure, protected the ecological environment with growth of the grassland area [48]. Grazing is the main production mode of beef, mutton, and milk, and the grain was replaced by forage to feed livestock in the QTP. Therefore, the grain for fodder in the QTP, one of the indirect grain consumptions, was lower than that of nationwide, the same with other parts of indirect grain consumption. Meanwhile, the yield per unit area and total grain yield were both increasing since 2003 as agricultural condition improved [49,50,51,52]. The yield per unit area increased from 4.13 to 4.80 t·hm^2^, total grain yield increased from 1.72 million ton to 2.10 million ton during 2003–2019 [36].

We transformed food into grain for they are grain-consuming products, except the vegetable and fruit, which simplified the process of food research and helps to plan land use. With the decrease of direct grain consumption and the slight increase of grain for fodder, the grain consumption will remain stable for a period time in the QTP. However, we have to know that the direct grain consumption cannot decrease all the time. It will remain stable when it is reduced to a certain amount. Additionally, the indirect grain consumption will increase with the development of the economy and food diversification in the future. Therefore, we need to survey the food consumption for a long time in the QTP.

Grain was also related closely to ecosystem protection and restoration programs. The per capita grain yield increased from 324.76 to 410.62 kg a^−1^ during 1980–1998 in China, since “Grain for Grain” was implemented in 2000. The per capita grain yield increased from 333.29 to 463.34 kg a^−1^ during 2003–2013 in China, the New Round of “Grain for Grain” was implemented since 2014 [53].

We hope this study can provide some implications for coordinating food production and implementation of ecosystem protection and restoration programs for other regions, particularly in pastoral and semipastoral areas. These areas distribute in Inner Mongolia, Ningxia, Xinjiang, Gansu, Qinghai, Tibet, Sichuan, and other provinces, and cover 264 counties, 4.05 million km^2^, where live 46.53 million people, most of them are Tibetan, Mongolian, Uygur, Hui, and Kazak [54], and their dietary structure is similar to that of the QTP. Pastoral areas and semipastoral areas are also important implementation areas of ecosystem protection and restoration programs, such as Grain for Green, the Three-North Shelterbelt System, and the “Two Screens and Three Belts” ecological security strategy [53,55,56]. The insight analysis of the grain consumption in these areas can effectively plan the land resource utilization mode, which will be of great significance to the specific implementation of ecosystem protection and restoration program.

## 5. Conclusions

Food and dietary structure of residents were corresponding to physical geographic and climatic environment. In the QTP, food consumption per capital was guaranteed at the well-off level from the perspective of nutritional demand since 2010. The conflict between food security and the ecosystem conservation can be managed without scarifying nature as the total grain consumption was stable since 2010, and the yield per unit area and total grain yield were both increasing since 2003 for agricultural condition improved in the QTP.

## Figures and Tables

**Table 1:** Comparison with the dietary pagoda of Chinese residents.

Types of Food	The Reasonable Dietary Pagoda of Chinese Residents	Food Consumption of QTP Residents	Result
Oil	25–30	38.16	Excessive
Milk	300	63.40	Insufficient
Pulse and nut	25–35	15.89	Insufficient
Meat and Poultry	40–75	97.86	Excessive
Aquatic products	40–75	8.90	Insufficient
Eggs	40–50	13.01	Insufficient
Vegetable	300–500	190.42	Insufficient
Fruit	200–350	69.77	Insufficient
Cereals and tubers	250–400	387.34	Meet

## Supplementary Materials

The following are available online at https://www.mdpi.com/article/10.3390/nu13113742/s1, Figure S1: Technology Roadmap, Table S1: Food Composition Tables.
