# Supplementary material for: Food and Grain Consumption Per Capita in the Qinghai–Tibet Plateau and Implications for Conservation
Source: Nutrients. 2021 Oct 23;13(11):3742. doi: 10.3390/nu13113742 (PMC8619228; doi:10.3390/nu13113742)
Supplement: Supplementary file 1 [file nutrients-13-03742-s001.zip › nutrients-1439708-supplementary.pdf]

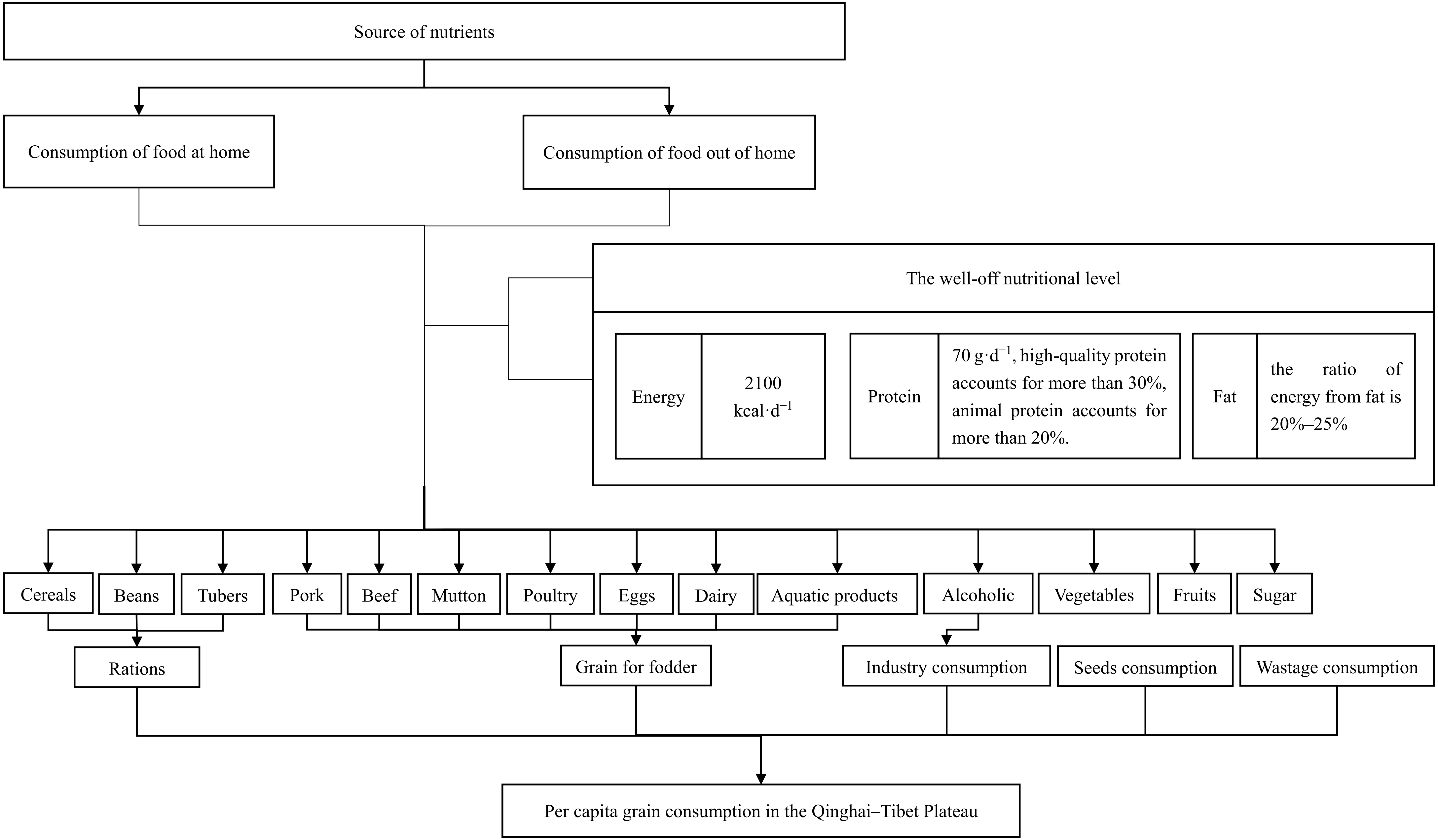

Figure. S1 Technology Roadmap

Table.S1 Food Composition Tables in Qinghai–Tibet Plateau /kg

Food types	Energy/kcal	Protein/g	Fat/g
1.Rations	3382.6	110.8	22.8
Cereals	3505.4	111.2	21.6
Fine grain	3516.8	116.1	19
Highland barley	3420	81	15
Wheat	3620	157	25
Rice	3460	79	9
Coarse grain	3459.5	91.65	31.7
Corn	3480	87	38
Sorghum	3600	104	31
Buckwheat	3370	93	23
Tubers	935	20	2
Potato	810	26	2
Sweet potato	1060	14	2
Beans	3792	321.9	130.1
Soybean	3900	350	160
Broad bean	3380	216	10
Peas	3340	203	11
2.Vegetable Oil	8990	0	999
3.Animal Oil	8820	0.6	974.2
Ghee	8600	1.5	944
Beef tallow	8980	0	997
Sheep fat	8950	0	990
Lard	8970	0	996
4.Animal meat products			
Meat			
Beef (cattle)	1600	200	87
Beef (yak)	1190	231	14
Mutton	1390	185	65
Pork	3310	151	301
Poultry	1442.5	199.4	80.1
Chicken	1450	203	67
Duck	240	155	197
Goose	2510	179	199
Aquatic Products	1037.4	163.04	34.26
Sea fish	1030	166	33
Freshwater fish	1130	166	52
5.Eggs	1439	129.75	91.7
Egg	1390	131	86
Duck	1800	126	130
Goose	1960	111	156
6.Dairy	642.2	30.66	35.87
Milk	650	33	36

	Ewe's milk	590	15	35
7.Vegetable		198	12.9	2.4
	Chinese cabbage	200	16	2
	Radish	160	7	1
	Eggplant	230	11	2
	Pepper	180	10	2
	Tomato	220	20	6
	Edible algae	900	18	1
8.Fruit		449.2	6	2
	Apple	530	4	2
	Pear	510	3	1
	Orange	580	10	2
	Banana	930	14	2
	Watermelon	310	5	3
	Muskmelon	260	4	1
	Brandy melon	230	6	1
	Cantaloupe	340	5	1
9.Sugar		3776	4.6	0
10.Alcohol		1842	0	0
